# Integrating Shared Decision‐Making, Motivational Interviewing, and Health Coaching in Obesity Care: A Stage‐Based Conceptual Framework for Sustainable Behavior Change

**DOI:** 10.1155/jobe/1398537

**Published:** 2026-11-06

**Authors:** Katarzyna Domosławska-Żylińska, Dominik Olejniczak, Dorota Włodarczyk

**Affiliations:** ^1^ Department of Health Promotion and System-Level Interventions National Institute of Public Health NIH-National Research Institute Warsaw 00-791 Poland; ^2^ Public Health Division Faculty of Health Science Medical University of Warsaw Warsaw 02-091 Poland wum.edu.pl; ^3^ Department of Health Psychology Medical University of Warsaw Warsaw 02-097 Poland wum.edu.pl

**Keywords:** behavior change, health coaching, motivational interviewing, obesity, shared decision-making

## Abstract

**Background:**

Obesity is a chronic, multifactorial disease that requires sustained modification of health behaviors. Although effective behavioral, pharmacological, and surgical treatments are available, long‐term weight loss maintenance remains challenging. Shared decision‐making (SDM), motivational interviewing (MI), and health coaching (HC) have been studied as patient‐centered approaches that may support communication, decision‐making, motivation, and engagement. However, their impact on behavioral and clinical outcomes, particularly sustained weight loss, varies considerably across studies. The aim of the review was to critically examine SDM, MI, and HC as complementary patient‐centered approaches to facilitating behavior change in obesity care and to propose a stage‐specific conceptual framework integrating these interventions across the clinical care pathway.

**Methods:**

A critical integrative narrative review was conducted using PubMed/MEDLINE, Scopus, and Web of Science. English‐language publications published between 2017 and 2026 addressing SDM, MI, or HC in adult obesity care were included. Evidence was synthesized using directed qualitative content analysis to compare theoretical foundations, mechanisms of action, clinical functions, and implementation considerations.

**Results:**

The reviewed approaches addressed distinct but interconnected phases of behavior change. SDM primarily supports informed treatment decisions aligned with patient values and preferences. MI facilitates the transition from ambivalence to action by strengthening autonomous motivation and readiness for change. HC focuses on the implementation and long‐term maintenance of health behaviors through goal setting, self‐monitoring, feedback, and self‐regulation strategies. Based on this synthesis, we propose a conceptual framework that maps the complementary primary functions of SDM, MI, and HC onto different, potentially overlapping phases of the behavior change process.

**Conclusions:**

SDM, MI, and HC should be viewed as complementary rather than competing approaches in obesity management. The proposed framework may help clinicians conceptualize how SDM, MI, and HC could complement one another in obesity care. Whether their integration improves patient engagement, treatment adherence, or long‐term weight management outcomes remains to be established empirically.

## 1. Introduction

Overweight and obesity have become one of the most pressing global public health challenges of the 21st century, affecting more than one billion people worldwide and nearly 60% of adults in the WHO European Region [1]. Obesity, classified as a chronic, complex, and multifactorial disease, is associated with an increased risk of noncommunicable diseases (including cardiovascular diseases, 13 types of cancer, Type 2 diabetes, and chronic respiratory diseases) and premature death [2]. Despite growing awareness of the problem and the availability of effective interventions, long‐term maintenance of weight loss remains limited, highlighting the importance of strategies aimed at sustainable modification of health behaviors [3].

Contemporary, evidence‐based approaches to obesity treatment include behavioral and psychological interventions, pharmacotherapy, and bariatric surgery. These methods serve as complementary tools to support the development of healthy eating patterns and increased physical activity, which are considered fundamental elements of the prevention and treatment of chronic diseases, including obesity [4, 5]. Also, in the era of growing popularity of obesity treatment with GLP‐1 drugs, it should be emphasized that the condition for their lasting effectiveness is the development of new eating behaviors [6]. However, the effectiveness of these interventions largely depends on the patient’s active involvement in the treatment process, including adherence to dietary recommendations, physical activity guidelines, medication regimens, and regular attendance at follow‐up visits. These processual and stage‐based theories suggest that the psychological tasks involved in behavior change may vary over time and that clinical support may, therefore, benefit from being adapted to patients’ current needs and readiness. However, the optimal timing and combination of SDM, MI, and HC in obesity care have not been established [7]. Behavior change is a complex process, and adherence to medical recommendations remains a significant challenge in all chronic diseases [4, 8]. Contemporary healthcare models are increasingly moving away from a paternalistic approach toward a patient‐centered model that emphasizes the role of autonomy and shared responsibility in the therapeutic process [9, 10]. Conventional interventions, focusing mainly on education, counseling, and promoting adherence, show limited effectiveness in achieving lasting behavioral change, pointing to a persistent gap between knowledge transfer and long‐term behavioral outcomes [11, 12]. From a behavioral science perspective, interventions that increase patient engagement, autonomy, and a sense of responsibility for health‐related goals are more likely to improve adherence and sustain behavioral change than interventions that rely primarily on providing information and behavioral advice [5, 13]. In response, numerous evidence‐based approaches to communication and behavior change have been developed, including shared decision‐making (SDM), motivational interviewing (MI), and health coaching (HC) [14, 15].

SDM is defined as a collaborative process in which doctors and patients make healthcare decisions together, integrating the best available scientific evidence with the patient’s values and preferences [14]. MI is a collaborative, person‐centered communication approach that is goal‐oriented in its focus on behavior change while respecting patient autonomy. It seeks to explore ambivalence and elicit the patient’s own motivations and reasons for change rather than impose a predetermined course of action [16]. HC focuses on supporting individuals in achieving health goals through structured goal‐setting, self‐monitoring, and feedback that reinforces self‐regulation and adherence to recommendations [17]. Although each of these approaches is supported by empirical evidence, they are typically implemented in isolation rather than as components of a coherent, stage‐based care pathway. Furthermore, their distinct mechanisms of action, clinical roles, and optimal timing in the behavior change process remain inadequately defined and are applied inconsistently in the treatment of obesity. The present conceptual perspective is informed by a synthesis of complementary stage‐based and processual models of behavior change. The transtheoretical model by DiClemente and Prochaska conceptualizes change as progressing through stages of readiness; Weinstein’s stage‐based model emphasizes transitions from risk awareness to behavioral engagement; whereas Schwarzer’s processual model distinguishes motivational and volitional phases responsible for action initiation and maintenance [18–20]. Together, these models suggest that different psychological tasks dominate at different phases of change and, therefore, may require different types of clinical support. Consequently, according to processual and stage‐based approaches to behavior change, the effectiveness of an intervention depends on matching its scope and function to the patient’s current stage of change. Although SDM, MI, and HC have each been associated with beneficial outcomes in different contexts, the potential value of their integration may depend not only on the approaches themselves, but also on how and when they are applied throughout obesity care. To address the lack of a structured, theoretically grounded integration of these approaches, we propose a comprehensive framework building on the health action process approach (HAPA) and self‐determination theory. This framework aligns SDM, MI, and HC with the patient’s evolving needs across the care pathway: SDM supports treatment‐related decision‐making, MI facilitates the exploration of ambivalence, motivation, and values, and HC assists in translating intentions into action and maintaining long‐term behaviors. These roles are not intended to represent mutually exclusive or fixed stages. Rather, the approaches may overlap and be revisited as patients’ goals, readiness, and support needs evolve. The aim of this review was to critically analyze SDM, MI, and HC as distinct yet complementary behavioral change interventions in the care of people with obesity. Through a comparative analysis of their mechanisms, scopes, and implementation potential, we propose a stage‐specific integrated framework that adapts each approach to key phases of the clinical pathway, from therapeutic decision‐making to long‐term behavior maintenance. The relatively widespread popularity of individual interventions in clinical settings and the interconnectedness between them appear to be factors facilitating the implementation of the integrated approach in practice.

## 2. Materials and Methods

This study was conducted as a critical‐integrative narrative review aimed at comparing and synthesizing selected approaches to behavior change in the context of obesity treatment. This methodological approach was deemed most appropriate due to the heterogeneity of the interventions analyzed, their differing theoretical foundations, and their varied roles in the clinical process. The literature review was conducted between March and May 2026, with the final literature search performed on May 5, 2026.

The analysis was limited to three approaches: SDM, MI, and HC, which are often recommended as strategies supporting patient engagement, autonomy, or treatment adherence. The differences between them and their congruence with behavior change models justify their selection as the subject of this analysis.

### 2.1. Literature Search Strategy

The search and selection process was designed to identify literature relevant to the conceptual comparison of SDM, MI, and HC in adult obesity care. The literature search was conducted in the PubMed/MEDLINE, Scopus, and Web of Science databases. We included English‐language publications released within the last 10 years (2017–2026) to reflect the current state of knowledge and clinical practice.

Combinations of keywords and MeSH terms were used, covering three main areas:

1. Population and clinical context: “obesity,” “overweight,” “weight management.”
2. Interventions: “shared decision making” OR “SDM”; “motivational interviewing” OR “MI”; “health coaching” OR “lifestyle coaching.”
3. Outcomes and behavioral mechanisms: “behavior change,” “adherence,” “patient engagement,” “self‐regulation,” “patient‐centered care.”

The logical operators AND/OR were used to combine the above categories.

 Inclusion criteria were defined as follows: publications on SDM, MI, or HC as the primary intervention; studies involving adult patients (≥ 18 years); studies conducted in the context of obesity; articles describing mechanisms of action, efficacy, clinical applications, or implementation aspects; and publications in English from the past 10 years.
 Exclusion criteria were defined as follows: studies involving pediatric populations, interventions not falling within the three analyzed approaches, publications focused exclusively on pharmacological or surgical treatment without a behavioral component. Following the application of the inclusion and exclusion criteria, a total of 29 peer‐reviewed articles met the eligibility criteria and were included in the final qualitative synthesis and conceptual mapping (Supporting Table 1).

### 2.2. Data Extraction and Qualitative Analysis

Data analysis was conducted using a qualitative approach based on directed content analysis, incorporating elements of comparative analysis. This approach was chosen due to the existence of a well‐established theoretical framework regarding behavioral change, which could serve as a starting point for the analytical structure while maintaining the ability to identify new, emergent categories. The analysis strictly followed the methodological principles established by Hsieh and Shannon (2005) for directed content analysis [51]. To guarantee methodological rigor and minimize potential bias, the literature screening, data extraction, and subsequent coding processes were conducted independently by two investigators (Domosławska‐Żylińska Katarzyna and Olejniczak Dominik). Any discrepancies or competing interpretations regarding thematic classification were resolved through discussion and consensus with a third senior researcher (Włodarczyk Dorota).

#### 2.2.1. Stages of the Analysis Process

##### 2.2.1.1. Preliminary Identification of the Analytical Framework (Deductive Preparatory Phase)

Based on theoretical and review literature concerning behavioral interventions, clinical communication, and implementation (including models of behavior change, patient‐centered care, and implementation science), a preliminary set of analytical categories was developed. These categories included, among others: the intervention’s objective, theoretical foundations, mechanisms of behavior change, the roles of the patient and clinician, the degree of directiveness, and the stage of the clinical pathway. The analysis was conducted in accordance with the directed content analysis approach, in which initial categories are defined based on existing theories and then iteratively modified during the course of data analysis [51].

##### 2.2.1.2. Iterative Literature Analysis (Inductive–Deductive Phase)

An in‐depth analysis of the selected publications was then conducted. During this phase, content was assigned to previously defined categories (deductive coding). Simultaneously, new, recurring themes that did not fit into the original structure were identified (inductive coding) and integrated into the revised coding scheme. On this basis, the categories were refined, expanded, or reorganized. For example, during the analysis, additional dimensions were identified, such as the potential for digitization, the intensity of intervention, or implementation constraints, which are not always explicitly present in classical theoretical models but are of significant clinical importance.

##### 2.2.1.3. Standardization and Comparison Across Interventions

In the next stage, a standardized analytical matrix was developed, enabling a direct comparison of the three interventions within the same categories. Each intervention (SDM, MI, and HC) was systematically described across all dimensions, allowing for the identification of similarities, differences, and areas of complementarity.

##### 2.2.1.4. Conceptual Synthesis and Integration of Results

The final phase involved synthesizing the results to develop an integrated conceptual framework. In this stage,

• The dominant functions of each intervention in the behavior change process were identified.
• They were mapped to the stages of the clinical pathway (decision–motivation–maintenance).
• Their potential for complementary application was analyzed.

The synthesis was interpretive in nature and grounded in existing theories of behavior change (including self‐determination theory, the transtheoretical model, and self‐regulation models), which enhanced its theoretical coherence and clinical utility.

## 3. Results

The interventions compared—SDM, MI, and HC—represent three complementary approaches with distinct primary functions within the broader behavior change process (Table 1 and Figure 1). SDM focuses primarily on the structure of the decision‐making process and serves as the starting point for clinical interaction, where aligning the therapeutic strategy with the patient’s values and preferences is key. In this approach, the central mechanism is not behavioral change itself, but rather the reduction of decision‐making uncertainty and the enhancement of alignment between the therapeutic choice and the patient’s individual health goals through processes such as risk communication, balanced presentation of treatment options, clarification of values, and deliberation on potential benefits and harms. In contrast, MI operates at the level of behavioral readiness, focusing on the dynamics of ambivalence and the transition from intention to action. This intervention serves as a “catalyst for change,” in which strengthening autonomous motivation, discovering resources that enable change, and activating the language of change play a fundamental role. Core mechanisms include reflective listening, eliciting “change talk,” exploring ambivalence, and developing discrepancy between current behaviors and long‐term goals. HC, on the other hand, covers the longest‐term time horizon and is aimed at stabilizing and maintaining changed behaviors. In this case, processes of self‐regulation, systematic monitoring, and the operationalization of health goals in the patient’s daily activities are of key importance. Typical strategies include structured goal setting, action planning, self‐monitoring, feedback loops, and problem‐solving focused on barriers to adherence. The common thread among all three approaches is a shift away from a paternalistic model toward empowering patients; however, they differ in the primary function of the intervention: SDM focuses primarily on the decision‐making stage, MI on the motivation stage, and HC on the implementation and maintenance stages.

**TABLE 1:** Comparison of approaches to support health behavior change (shared decision‐making, motivational interviewing, and health coaching) [21–50].

Dimension	Shared decision‐making (SDM)	Motivational interviewing (MI)	Health coaching (HC)
Primary objective	Collaborative, informed clinical decision‐making based on the integration of scientific evidence and patient values/preferences. Decision‐making [21–25]	Enhancing intrinsic motivation and readiness for behavior change through the resolution of ambivalence. Overcoming resistance and initiating action [28, 38]	Supporting the implementation and long‐term maintenance of health behavior change through goal structuring and self‐regulation. Maintenance of change [22, 28, 40–50]
Theoretical foundations	Patient autonomy model and evidence‐based medicine; decisional conflict theory; patient participation concepts [22–26]	Self‐determination theory; ambivalence theory; elements of the transtheoretical model of change [28–38]	Integrative approach based on behavior change theories: social cognitive theory, goal setting theory, self‐regulation [28, 40, 46, 47, 49, 50]
Behavioral change mechanisms	Reduction of decisional conflict; integration of patient preferences with medical information; increased perceived control [23, 24, 27]	Exploration and resolution of ambivalence; reinforcement of “change talk”; enhancement of autonomous motivation [28–38]	Goal setting; progress monitoring; habit reinforcement [28, 40–47, 49, 50]
Role of the patient	Active participant in the decision‐making process, cocreating the treatment plan according to personal values and preferences [22–27]	Individual exploring personal ambivalence and motivation, generating “change talk” [28–38]	Primary agent of behavior change, responsible for implementing and monitoring goals [22, 28, 40–47, 49, 50]
Role of the clinician	Expert and facilitator of the decision‐making process, providing information, and structuring options [22–26]	Nondirective facilitator of motivational change using autonomy‐supportive communication strategies [28–38]	Coach/mentor supporting the change process, monitoring progress, and reinforcing self‐regulation [28, 40–49]
Degree of directiveness	Moderate (shared): the clinician structures information and options, while decisions are cocreated with the patient [22, 25]	Low (nondirective but strategically goal‐oriented): the clinician guides the conversation toward exploring ambivalence and motivation without imposing decisions [28–38]	Low to moderate (facilitative): the clinician/coach does not prescribe solutions but actively structures goals, monitoring, and the change process [28, 40, 43–47, 49, 50]
Typical techniques	Decision aids; balanced presentation of evidence; risk communication (absolute vs. relative risk); values clarification; structured deliberation; presentation of treatment options [23–27]	MI core skills (OARS: open questions, affirmations, reflective listening, summaries); eliciting “change talk”; exploring ambivalence; discrepancy development; rolling with resistance [28–38]	SMART goal setting; action planning; self‐monitoring (e.g., diet, physical activity); feedback loops; reinforcement strategies; barrier identification and problem solving [28, 40–47, 49, 50]
Potential for digitalization and technological implementation	Moderate: online decision aids, risk visualization, and decision‐support tools feasible; limited full digital personalization [23, 27]	Moderate‐low: partial digitalization possible (teleconsultations, reflective chatbots), but full automation limited by the relational nature of MI [28–32, 36]	High: broad digitalization potential (mobile apps, wearables, coaching platforms, automated feedback loops); highly scalable in hybrid models [28, 40–43, 45–47, 49, 50]
Therapeutic relationship	Partnership‐based, grounded in shared decision‐making and a balance between information and values. Symmetrical relationship [21–23, 25]	Empathic, nondirective, autonomy‐focused, and centered on ambivalence exploration. An asymmetrical–facilitative relationship [28–36]	Supportive and mentoring relationship focused on developing patient competencies and self‐regulation. An asymmetrical–developmental relationship [22, 28, 40–44, 46–50]
Intervention intensity and durability of effects	Low‐moderate intensity/moderate durability: single or periodic decisional consultations; effects mainly related to treatment choice and alignment with patient values. Point‐based/episodic [23]	Moderate‐high intensity/moderate durability: requires time for ambivalence exploration and motivational enhancement; medium‐term effects dependent on ongoing support. Phase‐based [28–33, 39]	High intensity/high durability: long‐term process requiring regular contact, monitoring, and adaptation; highest potential for maintaining behavior change. Continuous/chronic [28, 40–50]
Required clinician/therapist competencies	Risk communication; presentation of evidence‐based information; facilitation of decision‐making; familiarity with decision‐support tools [22–24, 27]	Advanced communication skills (OARS); managing ambivalence; eliciting and reinforcing “change talk”; maintaining a nondirective stance [28–36]	SMART goal‐setting; behavioral planning; progress monitoring; supporting self‐regulation and adherence; often digital competencies [28, 40–50]
Implementation potential within healthcare systems	Moderate: can be improved through decision‐support tools and partial process standardization [22–24, 27]	Limited: highly dependent on clinician competence, time‐intensive sessions, and difficulty maintaining quality at scale [28–36]	High: possibility of delegation (e.g., coaches, digital platforms), automated monitoring, and scalable feedback systems [28, 40–47, 50]
Limitations and potential unintended consequences	Reduced effectiveness in patients with low health literacy; risk of information overload and decisional conflict; dependence on consultation time and communication skills [22–24, 27]	Strong dependence on training quality and intervention fidelity; risk of inconsistent application; limited effectiveness in patients not ready for change [28–36]	Risk of shifting responsibility excessively onto the patient; variability in coaching quality; attrition over time; dependence on technology in digital models [28, 40–50]
Stage of the clinical pathway	Treatment selection phase: used when patients are ready to make informed treatment decisions (e.g., diet, physical activity, pharmacotherapy, and bariatric surgery) [22–24]	Preparation and initiation phase: particularly effective in patients with ambivalence or low readiness for change; facilitates transition from contemplation to action [28–36]	Implementation and maintenance phase: applied after treatment decisions to reinforce healthy behaviors, monitor progress, and prevent relapse [28, 40–50]
Optimal clinical application in obesity care	Patients with established readiness for change requiring selection among treatment strategies (e.g., lifestyle intervention vs. pharmacotherapy vs. bariatric surgery, including patients with type 2 diabetes). Key moment: therapeutic decision‐making in primary or specialist care [22–24]	Patients with ambivalence, low motivation, or previous unsuccessful weight loss attempts; especially effective during the contemplation–preparation phase. Key objective: initiation of action [28–36]	Patients who have already initiated lifestyle modification; particularly useful for long‐term weight loss maintenance, prevention of weight regain, and sustainable habit change [22, 28, 40–50]

*Note:* The stages presented in Table 1 indicate the primary clinical function of each approach rather than exclusive periods of application. SDM, MI, and HC may be used concurrently or revisited throughout obesity care, depending on the patient’s preferences, readiness for change, treatment response, and evolving support needs. Durability and implementation assessments are conceptual interpretations based on theory and available evidence, not results of direct comparative trials or clinical rankings. Evidence strength for weight loss and long‐term maintenance varies by approach.

**FIGURE 1:**
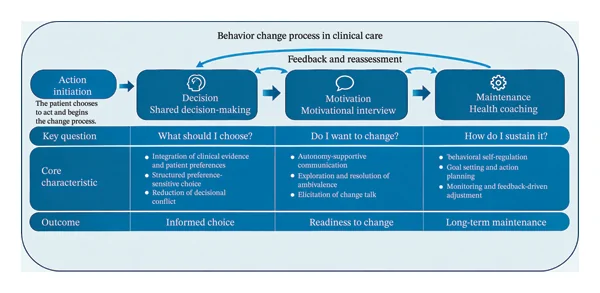
Behavior change process in clinical care (own source).

## 4. Discussion

This review highlights that the treatment of obesity requires a shift from isolated behavioral interventions to an integrated, developmentally sensitive communication model that combines decision‐making, motivation, and long‐term behavior maintenance. Although SDM, MI, and HC are independently evidence‐based, their application remains fragmented, limiting their cumulative impact on long‐term outcomes in obesity treatment. The clinical utility of our proposed framework is particularly evident when applied to contemporary challenges in obesity care, such as the rapid integration of pharmacological treatments. For instance, a clinician and patient may initially utilize SDM to deliberate on evidence‐based options, selecting between a primary lifestyle intervention and the addition of a GLP‐1 receptor agonist. Such decisions should take into account the distinct therapeutic contributions of lifestyle interventions, pharmacotherapy, and metabolic/bariatric surgery, as well as the patient’s preferences, clinical needs, and treatment goals. Once the treatment modality is established, however, patients may experience ambivalence regarding long‐term pharmacological treatment, concerns about adverse effects, or uncertainty about whether to continue medication. In these situations, SDM may need to be revisited to reassess the benefit–risk balance, address the patient’s concerns, and jointly determine the most appropriate course of action. MI can then help explore ambivalence, elicit the patient’s own motivations, and strengthen autonomous motivation for the agreed treatment plan. Subsequently, as the action phase commences, HC provides the necessary volitional architecture, such as SMART goal structuring and self‐monitoring feedback loops, to sustain these newly acquired health behaviors and support long‐term adherence. Importantly, behavioral support should be understood as a component of comprehensive obesity treatment, rather than a prerequisite for the therapeutic efficacy of GLP‐1 receptor agonists. Although the development of sustainable eating and other health‐related behaviors may complement pharmacotherapy and support long‐term weight management, the therapeutic effects of pharmacological treatment should not be considered contingent upon the successful adoption of new eating behaviors. This integrated framework, therefore, aims to align behavioral support with the patient’s chosen treatment modality, while recognizing that treatment outcomes reflect the contributions of different therapeutic approaches and that ongoing care may require reassessment and adaptation over time.

SDM primarily supports the quality of treatment‐related decision‐making by integrating clinical evidence with patients’ values and preferences. Its potential benefits should be distinguished from effects on subsequent health behaviors and clinical outcomes. Evidence concerning SDM and decision aids supports their role in facilitating informed, preference‐sensitive decisions, although improvements in decision quality do not necessarily translate into sustained behavioral change or long‐term weight loss. The extent to which additional motivational or behavioral support improves these downstream outcomes depends on the intervention and clinical context [52, 53]. These results confirm the interpretation of SDM as a layer of decision‐making architecture rather than a strategy for sustaining behaviors.

MI plays a key role in resolving ambivalence and strengthening intrinsic motivation, particularly among individuals with low readiness to change [37]. Evidence on MI in obesity care is heterogeneous. Improvements in motivational processes or engagement should not be interpreted as equivalent to a consistent additional effect on body weight. In particular, findings from individual trials and reviews need to be interpreted in light of the intervention design, comparator, follow‐up duration, and clinical context. In the study by Michalopoulou M (2022), the additional weight benefit associated with the intervention was limited, suggesting that improvements in motivational processes do not necessarily translate into substantial additional weight loss [39]. Other research indicates the validity of using MI for weight loss in primary care [38]. The theoretical foundations of MI, based on self‐determination theory, emphasize supporting autonomy as a key factor in behavioral sustainability [54]. MI is primarily concerned with exploring ambivalence and supporting autonomous motivation, rather than providing a standardized system for ongoing behavioral monitoring. Its duration and timing depend on the clinical context and the patient’s needs. Although MI may be particularly useful when patients are considering or initiating behavior change, it may also be revisited during follow‐up when ambivalence, difficulties with adherence, or changing treatment goals arise. In the proposed framework, MI is, therefore, presented as a flexible source of motivational support rather than an intervention confined to a single transition between decision and action.

HC uses goal setting, self‐monitoring, feedback, and problem‐solving to support the implementation of health‐related goals and behavioral self‐regulation [55]. However, evidence for its effects on weight loss and long‐term weight maintenance remains uncertain. A systematic review and meta‐analysis of HC for weight loss reported findings that should be interpreted in light of the limited certainty of the available evidence [50]. Thus, although coaching may offer practical support for implementing and maintaining behavior change, its effects on weight‐related clinical outcomes should not be assumed to be consistent or substantial across settings. Intervention intensity, continuity, patient engagement, and attrition may also influence observed outcomes [56, 57]. Importantly, the role of MI may extend beyond the initial phase of motivational engagement and may be revisited during long‐term follow‐up alongside HC. While HC can provide ongoing practical support through goal setting, self‐monitoring, and problem‐solving, MI may be useful when patients experience renewed ambivalence, difficulties adhering to treatment recommendations, or changes in their readiness to sustain health‐related behaviors. This complementary use may be particularly relevant during postoperative bariatric follow‐up, when patients’ support needs can evolve over time. However, reduced participation in coaching sessions should not be interpreted as evidence of declining motivation unless motivation has been directly assessed. Rather, it may indicate a need to explore potential barriers to engagement and reassess the type of support required.

Empirical studies have examined motivational and decision‐support approaches in different clinical contexts, although these studies have not directly evaluated the integrated framework proposed here. For example, a pragmatic randomized trial in patients with chronic noncancer pain compared MI combined with cognitive behavioral therapy with SDM visits and found no significant between‐group differences in opioid dose reduction or functional outcomes [58]. Similarly, in patients with recent fractures requiring antiosteoporosis medication, a multicomponent intervention combining SDM (including a patient decision aid) and MI within fracture liaison services improved the quality of the decision‐making process but did not significantly affect long‐term medication persistence, adherence, or clinical outcomes compared with usual care [59]. In contrast, implementation data from a large HC program incorporating MI demonstrated high initial participation rates, suggesting good acceptability of MI‐based coaching; however, sustained engagement across multiple sessions was substantially lower, highlighting challenges in maintaining long‐term behavioral support even when motivational techniques are embedded in coaching frameworks [60]. Taken together, these findings indicate that SDM, MI, and HC are each associated with specific potential benefits, such as improved decision quality, increased engagement, or enhanced readiness to change. However, their combined application has not yet been structured into a coherent, stage‐matched model spanning decision‐making, motivation, and long‐term maintenance.

When analyzing approaches that support decision‐making and changes in health behaviors, it is essential to consider key factors that influence their effectiveness, such as patients’ health literacy and clinicians’ communication skills. Low health literacy is associated with poorer understanding of risk information, less engagement in SDM, and lower adherence to behavioral recommendations [61–63]. Similarly, clinicians’ communication skills significantly influence patient treatment outcomes. Effective risk communication, empathy, and autonomy‐supportive communication have been linked to better adherence to behavioral recommendations and satisfaction with treatment [64, 65]. These findings underscore that the success of SDM, MI, and HC does not depend solely on the intervention model itself, but also on systemic communication competencies and patient skills.

### 4.1. Limitations and Future Directions

Several limitations of this review should be acknowledged. First, the available evidence on SDM, MI, and HC is characterized by considerable conceptual and terminological heterogeneity. In particular, noticeable inconsistencies exist in the definitions, scope, and operationalization of HC and MI across studies. The lack of uniform terminology and standardized intervention descriptions limited the comparability of findings and may have resulted in the omission of potentially relevant studies that did not explicitly use the selected terminology. Second, this study was conducted as a narrative integrative review rather than a systematic review or meta‐analysis. Consequently, the synthesis was based on an interpretative comparison of the literature, which may introduce a degree of subjectivity in the selection, categorization, and interpretation of evidence. Third, the proposed framework is conceptual in nature and has not yet been empirically validated. The proposed mapping of SDM, MI, and HC onto different primary functions within the behavior change process is conceptual and has not been empirically validated as an integrated model. Although informed by established theoretical models and evidence concerning the individual approaches, the framework’s proposed relationships, optimal timing, and potential added value remain hypothetical until evaluated in prospective clinical studies. Finally, the review focused exclusively on adult populations with overweight and obesity and did not address potential differences related to age, socioeconomic status, cultural background, or healthcare system characteristics, all of which may influence the effectiveness and implementation of the proposed interventions.

Future research should focus on empirically evaluating the proposed integrated framework in clinical practice. In particular, prospective studies are needed to examine whether a stage‐specific model combining SDM, MI, and HC can improve long‐term weight management outcomes, treatment adherence, patient engagement, and the maintenance of health behavior changes compared with the isolated use of these interventions. It would be particularly important to verify which elements and techniques from each stage are most effective and which durations of the respective stages would be most beneficial. Another aspect would be to examine the extent to which this integrated approach remains effective when obesity care is expanded to include additional, specific behavioral goals, and when the intervention encompasses a series of parallel processes that unfold at different rates. Further research should also aim to establish greater conceptual clarity and standardization regarding the definitions, competencies, and core components of HC and MI. Such standardization would facilitate comparison across studies and support the development of implementation guidelines. Additionally, implementation studies are needed to assess the feasibility, cost‐effectiveness, and scalability of the proposed model in different healthcare settings, including primary care, specialist obesity clinics, and digitally supported care pathways. Particular attention should be given to the role of digital technologies and artificial intelligence–based tools in supporting long‐term behavior maintenance and personalized patient support.

## 5. Conclusions

• SDM, MI, and HC should not be viewed as competing approaches but rather as complementary elements of a long‐term process of engaging patients with obesity, which strengthens their empowerment and responsibility for their own health.
• Effective treatment of obesity requires tailoring the type of intervention to the patient’s needs, capabilities, and stage of behavioral readiness, taking into account measures that support the development of independence and lasting changes in health behaviors.
• Clinicians’ communication skills and their ability to support patients in understanding the treatment process and making informed decisions are significant individual factors influencing the effectiveness of therapeutic interventions.
• The proposed framework provides a conceptual basis for coordinating decision‐making, motivational support, and behavioral self‐regulation in obesity care. Its clinical utility, including the optimal timing and combination of the three approaches, requires prospective evaluation.

## Author Contributions

Katarzyna Domosławska‐Żylińska and Dominik Olejniczak: study concept; Katarzyna Domosławska‐Żylińska and Dominik Olejniczak: data collection; Katarzyna Domosławska‐Żylińska, Dominik Olejniczak,, and Dorota Włodarczyk: data analysis; Katarzyna Domosławska‐Żylińska, Dominik Olejniczak, and Dorota Włodarczyk: data interpretation; Katarzyna Domosławska‐Żylińska: manuscript drafting; Dominik Olejniczak and Dorota Włodarczyk: manuscript supervision.

## Funding

The research was carried out as part of research tasks conducted by the National Institute of Public Health PZH–National Research Institute, financed by the Minister of Health under contract no. NIZP PZH‐PIB/2026/1094/113 and the Medical University of Warsaw (grant no. 2MB/2026/RDW/N/Z3).

## Disclosure

All authors have read the final version of the manuscript and approved its content.

## Ethics Statement

The authors have nothing to report.

## Conflicts of Interest

The authors declare no conflicts of interest.

## Supporting Information

Additional supporting information can be found online in the Supporting Information section.

## Supporting information


**Supporting Information** Table 1 List of articles included in the literature review.

## Data Availability

All data generated or analyzed during this study are included in this published article.
