# Supplementary material for: Integrating Shared Decision‐Making, Motivational Interviewing, and Health Coaching in Obesity Care: A Stage‐Based Conceptual Framework for Sustainable Behavior Change
Source: J Obes. 2026 Nov 6;2026:1398537. doi: 10.1155/jobe/1398537 (PMC13741395; doi:10.1155/jobe/1398537)
Supplement: Supplementary file 1 [file JOBE-2026-1398537-s001.docx]

Supplementary

Table 1 List of articles included in the literature Review

|  | Authors | title | Journal/book/ | year |
| --- | --- | --- | --- | --- |
| 1 | M. Hawke, L. Sweet, J. Considine | Shared Decision-Making and Body Mass Index in Australian Antenatal Care: An Exploratory OPTION12 Evaluation | Health expectations : an international journal of public participation in health care and health policy 27, no. 6 doi:10.1111/hex.70107. | 2024 |
| 2 | K. A. Gudzune, L.M. Kaplan, S. Kahan, et al. | Weight-Reduction Preferences Among OBSERVE Study Participants With Obesity or Overweight: Opportunities for Shared Decision-Making. | Endocrine practice : official journal of the American College of Endocrinology and the American Association of Clinical Endocrinologists 30,no.10: 917-926. doi:10.1016/j.eprac.2024.06.009 | 2024 |
| 3 | C. Nnonyelu, C. Onyebuchi, H.N. Okeke, et al. | Shared Decision-Making and Obesity Management Across Healthcare Models in the United States: A Narrative Review | Medtigo J Med. 3, no. 2:e30623219. | 2025 |
| 4 | L.M. Schumacher, S.B. Bass, J. Ard, et al. | The New Obesity Treatment Landscape: Challenges and Opportunities to Promote Shared Decision-Making in People With Obesity and Type 2 Diabetes. | Diabetes care 49, no. 6: 906-915. doi:10.2337/dci25-0094. | 2026 |
| 5 | J.R. Imbus, and L.M. Funk | Shared decision-making in obesity treatment | Morton J, Brethauer S, DeMaria E, Kahan S, Hutter M, eds. Quality in Obesity Treatment. Cham, Switzerland: Springer; 17. doi:10.1007/978-3-030-25173-4_17. | 2019 |
| 6 | H. Bomhof-Roordink, F.R. Gärtner, A.M. Stiggelbout, et al. | Key components of shared decision making models: a systematic review | BMJ open 9, no.12: e031763. doi:10.1136/bmjopen-2019-031763 | 2019 |
| 7 | Y.T. Chu, R.Y. Huang, T.T. Chen, et al. | Effect of health literacy and shared decision-making on choice of weight-loss plan among overweight or obese participants receiving a prototype artificial intelligence robot intervention facilitating weight-loss management decisions | Digital health 8: 20552076221136372. doi:10.1177/20552076221136372 | 2022 |
| 8 | J.J. Rodriguez-Cristobal, C. Alonso-Villaverde, J.M. Panisello, et al. | Effectiveness of a motivational intervention on overweight/obese patients in the primary healthcare: a cluster randomized trial. | BMC family practice 18, no.1: 74. doi:10.1186/s12875-017-0644-y | 2017 |
| 9 | J. Andino, J. Park-Mroch, S.L. Francis, et al. | A randomized controlled trial of a community-based obesity intervention utilizing motivational interviewing and community resource mobilization for low-income families: Study protocol and baseline characteristics. | Contemporary clinical trials 112: 106626. doi:10.1016/j.cct.2021.106626 | 2022 |
| 10 | E. Centis, Elena, M.L. Petroni, V. Ghirelli,et al. | Motivational Interviewing Adapted to Group Setting for the Treatment of Relapse in the Behavioral Therapy of Obesity. A Clinical Audit. | Nutrients 12, no.12:3881. doi:10.3390/nu12123881 | 2020 |
| 11 | R. D. Barnes, V. Ivezaj, S. Martino, et al. | Examining motivational interviewing plus nutrition psychoeducation for weight loss in primary care | Journal of psychosomatic research 104: 101-107. doi:10.1016/j.jpsychores.2017.11.013 | 2018 |
| 12 | H. Makin, A. Chisholm, V. Fallon, et al. | Use of motivational interviewing in behavioural interventions among adults with obesity: A systematic review and meta-analysis | Clinical obesity 11, no. 4: e12457. doi:10.1111/cob.12457 | 2021 |
| 13 | V. Moizé, Y. Graham, X.R. Salas,et al. | Motivational Interviewing (MI) in Obesity Care: Cultivating Person-Centered and Supportive Clinical Conversations to Reduce Stigma: A Narrative Review. | Obesity science & practice 11, no.1: e70057. doi:10.1002/osp4.70057 | 2025 |
| 14 | K. Szczekala, B. Ślusarska, and A. Goś. | Motivational interviewing in obesity reduction | Medical Studies/Studia Medyczne 33, no.1:73-80. | 2017 |
| 15 | M. Freshwater, et al. | Behavior, motivational interviewing, eating disorders, and obesity management technologies: An Obesity Medicine Association (OMA) Clinical Practice Statement (CPS) 2022 | Obesity Pillars 2:100014 .https://doi.org/10.1016/j.obpill.2022.100014. | 2022 |
| 16 | L. David, and S.E. Cassin | Motivational interviewing for behavioral and lifestyle changes in severe obesity | In: Cassin S, Hawa R, Sockalingam S, eds. Psychological Care in Severe Obesity: A Practical and Integrated Approach. Cambridge, UK: Cambridge University Press:79-104. doi:10.1017/9781108241687.006. | 2018 |
| 17 | I. Tsampoula | The role of motivational interviewing in the management of obesity in adults | World J Adv Res Rev. 24, no. 2:1421-1428. doi:10.30574/wjarr.2024.24.2.2831 | 2024 |
| 18 | M. Michalopoulou, A.E. Ferrey, G. Harmer, et al. | Effectiveness of Motivational Interviewing in Managing Overweight and Obesity : A Systematic Review and Meta-analysis | Annals of internal medicine 175, no. 6: 838-850. doi:10.7326/M21-3128 | 2022 |
| 19 | H.S.J. Chew, N.N. Rajasegaran, Y.H. Chin,et al. | Effectiveness of Combined Health Coaching and Self-Monitoring Apps on Weight-Related Outcomes in People With Overweight and Obesity: Systematic Review and Meta-analysis. | Journal of medical Internet research 25: e42432. doi:10.2196/42432 | 2023 |
| 20 | S. Driscoll, G. Meyerowitz-Katz, G. Ahlenstiel, et.al. | Efficacy of Telephone Health Coaching Integration with Standard Multidisciplinary Care for Adults with Obesity Attending a Weight Management Service: A Pilot Study | Nutrients 13, no. 11: 4078. <https://doi.org/10.3390/nu13114078> | 2021 |
| 21 | N.I. Heredia, M. Lee, K.O. Hwang, et al. | Health coaching to encourage obese adults to enroll in commercially-available weight management programs: The path to health study | Contemporary clinical trials 83: 1-9. doi:10.1016/j.cct.2019.06.006 | 2019 |
| 22 | M.R. Jay, S. Wittleder, S. Vandyousefi, et al. | A Cluster-Randomized Study of Technology-Assisted Health Coaching for Weight Management in Primary Care | Ann Fam Med. 22, no 5:392-399. doi: 10.1370/afm.3150. | 2024 |
| 23 | C. van Rinsum, S. Gerards, G. Rutten, et al. | The Coaching on Lifestyle (CooL) Intervention for Overweight and Obesity: A Longitudinal Study into Participants' Lifestyle Changes. | International journal of environmental research and public health 15, no. 4: 680. doi:10.3390/ijerph15040680 | 2018 |
| 24 | K.E. Johnson, M.K. Alencar, K.E. Coakley, et al. | Telemedicine-Based Health Coaching Is Effective for Inducing Weight Loss and Improving Metabolic Markers. | Telemedicine journal and e-health : the official journal of the American Telemedicine Association 25, no. 2: 85-92. doi:10.1089/tmj.2018.0002 | 2019 |
| 25 | J.M. Silberman, M. Kaur, J. Sletteland, et al. | Outcomes in a digital weight management intervention with one-on-one health coaching. | PloS one 15, no. 4: e0232221. doi:10.1371/journal.pone.0232221 | 2020 |
| 26 | S. Wittleder, A. Ajenikoko, D. Bouwman, et al. | Protocol for a cluster-randomized controlled trial of a technology-assisted health coaching intervention for weight management in primary care: The GEM (goals for eating and moving) study. | Contemporary clinical trials 83: 37-45. doi:10.1016/j.cct.2019.06.005 | 2019 |
| 27 | H.D. Kleine, L.A. McCormack, A. Drooger, et al. | Barriers to and Facilitators of Weight Management in Adults Using a Meal Replacement Program That Includes Health Coaching | Journal of primary care & community health 10: 2150132719851643. doi:10.1177/2150132719851643 | 2019 |
| 28 | R.R. Suminski, T. Leonard, I. Obrusnikova, et al. | The Impact of Health Coaching on Weight and Physical Activity in Obese Adults: A Randomized Control Trial. | American journal of lifestyle medicine 18, no. 2: 233-242. doi:10.1177/15598276221114047 | 2022 |
| 29 | S.S. Sieczkowska, A.P. de Lima, P.A. Swinton, et al. | Health Coaching Strategies for Weight Loss: A Systematic Review and Meta-Analysis. | Advances in nutrition (Bethesda, Md.) 12, no. 4: 1449-1460. doi:10.1093/advances/nmaa159 | 2021 |
